# Now or Never!

**Published:** 1904-08

**Authors:** 


### TEXAS MEDICAL JOURNAL.

AUSTIN, TEXAS.

A MONTHLY JOURNAL OF MEDICINE AND SURGERY.

EDITED AND PUBLISHED BY
F. E. DANIEL, M. D.
Mrs. F. E. DANIEL, Managing Editor.

Published Monthly at Austin, Texas. Subscription price $1.00 a year in advance.
Eastern Representative: John Guy Monihan, St. Paul Building, 220 Broadway, New York City.
Official organ of the West Texas Medical Association, the Houston District Medical Association, the Austin District Medical Society, the Brazos Valley Medical Association, the Galveston County Medical Society, and several others.

### NOW OR NEVER!

The Texas Legislature will meet in January. In November Senators and Representatives are to be elected. Let my readers refresh their memories by reading over the editorials and the several bills in January, February, March and April, 1903, numbers of the "Red Back" and fix well in their minds the men who defeated our efforts both for sanitary legislation, vital statistics and amendment to the medical practice act, and if these men are candidates for re-election, move heaven and earth to defeat them, or convert them to reason and justice.

Now that the medical profession has been organized from the ground up, and every county society has or should have a legislative committee, it should make its influence felt. The medical profession is conscientiously working in the cause of sanitation and the protection of the public health, and, numbering as they do about six thousand regular physicians—about one-half of whom are enrolled the first year of organization—they constitute a factor in politics to be reckoned with. Concert of action on their part will elect or defeat any candidate for the Legislature.

The duty of these legislative committees, in addition to seeing that the medical practice act is enforced (so far as it can accomplish anything), should be to secure in advance from candidates pledges of support of the State Medical Association bills for sanitation and a State Board of Health to execute it, vital statistics and a registrar, and regulating the practice of medicine, if possible. Really, the last mentioned comes strictly under the province of "sanitation," meaning measures for the protection of health, for the ignorant "doctor"—the drugless terrors unwisely exempted by the State from the necessity of being licensed, is as great a menace to the public health as any disease or disease-producing condition, and that obnoxious, unjust, unreasonable and *unconstitutional* clause which *shows preference** to them, should and must be eliminated. Every one permitted by the State to *practice medicine* should toe the same mark. These drugless ones are practicing medicine. They claim to be educated in all branches except materia medica and therapeutics, and to hold diplomas from colleges. Why, then, in the name of reason, should they not be amenable to the law?

Now, here is what I propose: Remodel the existing law which exempts all who do not give drugs,—so as to make it apply to all who undertake to treat disease,—except the Christian Science cranks. Let them alone. Let them pray over chronic cases, but under severe penalty prohibit them from undertaking to deal with wounds, injuries, contagious or infectious diseases, or, in fact, any acute cases. They do not pretend to be educated in any of the branches of medicine, and to require them to be examined would be tantamount to prohibition, and at once the cry would be raised, "religious persecution." That is what beat us; let's fix them so they can do no harm, and let it go at that.

Now let all the county societies go to work; send out their members to "interview" candidates, and get in their work in the interest ol humanity. I especially call attention to the March, 1903, number of the "Red Back." Let the county societies have the letters there published printed and distributed. It will enlighten many well-meaning candidates,—will open their eyes. I know by conversation with intelligent members that they are deluded by the belief that if a man *gives no medicine* he is not practicing medicine! The courts have ruled in numerous cases that the drugless ones are practicing medicine within the meaning of the law. But for the clause in our law which especially exempts them, they could be gotten at.

AS AN INCENTIVE to county societies and an illustration and

*The Constitution says: "The State shall have the power to regulate the practice of medicine, but no preference shall be shown to any school of medicine."

example of the kind of work to be done between now and November, I publish below an extract from a letter from a member of the State Association's Legislative Committee, and an ex-Councilor (suppressing names):

——————, August 1, 1904.

*Dr. F. E. Daniel, President State Medical Association of Texas, Austin, Texas.*

DEAR DOCTOR: We have just held our primary elections, and, as far as I have heard, we swept out every man that voted against the amendments we wanted last year. We elected —————— of this place to the State Senate, and he is one of the ablest lawyers in the State, and is pledged to any measures we want. He says he owes his election to the efforts of the doctors of this district, and that he will make any measure we want go through the Senate, or know why it fails.

The two representatives of this county are both strong men and will help up all they can. I went over to the Senatorial District just north of us and helped to defeat Senator ——————, who voted with the drugless doctors in the last Legislature. If you have any literature that will help to show the wrong of exempting the drugless doctors, please send it to me so that I can supply the men to whom we can look for help in this part of the State.

Yours truly,

——————

*I sent it.* Go thou and do likewise.

